# Lithotritie

**Published:** 1829-05

**Authors:** 


					412 HOSPITAL REPORTS.
LITHOTRITIE.
Case in which the Operation of Lithotritie was successfully "per-
formed by Robert Liston, Esq. one of the Surgeons of the
Royal Infirmary of Edinburgh, Lecturer on Surgery, &c.
Andrew Leeqhman, aged seventy, was admitted into the Royal
Infirmary on the 10th November, 1828. He stated that, for five
months past, he had been labouring under all the symptoms of
stone in the bladder. On sounding him, a stone was distinctly
felt. As he had a great aversion to being cut, and as his urine
seemed to indicate a diseased state of the bladder, it was thought
advisable to break down the stone, in preference to the usual
operation.
On 13th November, a solution of opium having been injected
into the bladder, Mr. Liston introduced Civiale's instrument, but,
owing to the restlessness of the patient and the irritable st^te of
the bladder, did not succeed in grasping it completely. Several
small portions of stone, however, came away in the fangs of the
instrument, and during the night. He suffered no inconvenience
from the operation.
On the 15th, he passed a barleycorn incrusted with calcareous
matter. On the 16th, a piece of straw with the same incrustation.
He complained of pain in the testicles. On the 18th, a small
abscess having formed in the scrotum it was opened.
The instrument was again introduced on the 25th. The stone
was fairly laid hold of, but was so soft that it was crushed by the
instrument; on withdrawing which several fragments of seeds
were found adhering. He now confessed that, while reaping dur-
ing the last harvest, he had introduced a number of barleycorns
into his urethra, but would not say for what purpose.
The patient had repeated attacks of retention of urine after last
operation, from the larger portions of stone lodging in the urethra.
He passed in all thirteen fragments, having entire barleycorns for
their nucleus, besides a much greater number having only small
pieces of the beards. He had now little pain, and the quantity of
mucus in his urine was inconsiderable. He was sounded several
times, and, as nothing was felt in his bladder, he was dismissed
cured on the 16th December, 1828.*
Edinburgh Med. Journal, April.

				

